# Impact of Immunosuppression on the Metagenomic Composition of the Intestinal Microbiome: a Systems Biology Approach to Post-Transplant Diabetes

**DOI:** 10.1038/s41598-017-10471-2

**Published:** 2017-08-31

**Authors:** M. Bhat, E. Pasini, J. Copeland, M. Angeli, S. Husain, D. Kumar, E. Renner, A. Teterina, J. Allard, D. S. Guttman, A. Humar

**Affiliations:** 1 Multi Organ Transplant Program, University Health Network, Toronto, Canada; 2 Division of Gastroenterology and Hepatology, Department of Medicine, University Health Network, Ontario, Canada; 3 Division of Gastroenterology and Hepatology, Department of Medicine, University of Toronto, Toronto, Canada; 4 Centre for the Analysis of Genome Evolution and Function, University of Toronto, Toronto, Canada; 5 Division of Infectious Diseases, Department of Medicine, University Health Network, Department of Medicine University of Toronto, Toronto, Canada; 6 Division of Infectious Diseases, Department of Medicine University of Toronto, Toronto, Canada; 7 Department of Cell and Systems Biology, University of Toronto, Toronto, Canada

## Abstract

Solid organ transplantation (SOT) outcomes have continued to improve, although long-term use of immunosuppressants can lead to complications such as diabetes, compromising post-transplant outcomes. In this study, we have characterized the intestinal microbiome (IM) composition at the metagenomic level in the context of hyperglycemia induced by immunosuppressants. Sprague-Dawley rats were subjected to doses of tacrolimus and sirolimus that reliably induce hyperglycemia and an insulin-resistant state. Subsequent exposure to probiotics resulted in reversal of hyperglycemia. 16S rRNA and metagenomic sequencing of stool were done to identify the bacterial genes and pathways enriched in immunosuppression. Bacterial diversity was significantly decreased in sirolimus-treated rats, with 9 taxa significantly less present in both immunosuppression groups: *Roseburia, Oscillospira, Mollicutes, Rothia, Micrococcaceae, Actinomycetales and Staphylococcus*. Following probiotics, these changes were reversed to baseline. At the metagenomic level, the balance of metabolism was shifted towards the catabolic side with an increase of genes involved in sucrose degradation, similar to diabetes. Conversely, the control rats had greater abundance of anabolic processes and genes involved in starch degradation. Immunosuppression leads to a more catabolic microbial profile, which may influence development of diabetes after SOT. Modulation of the microbiome with probiotics may help in minimizing adverse long-term effects of immunosuppression.

## Introduction

Survival after solid organ transplantation (SOT) has improved over time. However, outcomes are compromised by long-term complications such as diabetes, obesity, and de novo/recurrent fatty liver disease that put patients at higher risk of cardiovascular disease^1^. In particular, new-onset diabetes mellitus after transplant (NODAT) occurs in a large proportion of patients, with estimates ranging from 17–53%^1, 2^. Immunosuppressive therapy is a key risk factor for NODAT, in addition to age, family history, and ethnicity^1^. Insulin secretion, insulin resistance and direct toxic effect on pancreatic B-cells are the proven mechanisms through which immunosuppressants foster a hypeglycemic state^3, 4^.

The literature in non-transplant patient populations suggests that the intestinal microbiome (IM) plays a role in the development of diabetes, obesity and NAFLD^5^. Two large metagenome-wide association studies revealed a decrease in butyrate-producing bacteria such as *Roseburia* and *Faecalibacterium prausnitzii* in the IM of diabetic patients^6, 7^. The IM in obese individuals is more efficient at energy harvest from the diet^8, 9^. The transferability of the diabetic and obese phenotypes has been confirmed in animal models^10^. Cause-and-effect has also been demonstrated with fecal microbiota transplantation. A randomized controlled trial of fecal transplantation from lean donors to insulin-resistant males with metabolic syndrome demonstrated improved peripheral insulin sensitivity^11^. This occurred in conjunction with enhanced intestinal microbial diversity and increased levels of butyrate-producing bacteria such as *Roseburia* ^11^. Oral butyrate has been shown to decrease insulin resistance and increase energy expenditure *in vivo* ^12^. Butyrate-producing bacteria play a key role in not only maintaining intestinal health, but also in promoting insulin sensitivity systemically^13^.

Given the demonstrated ability of the IM to promote an insulin-resistant state, and the long-term metabolic complications causing significant morbidity in SOT recipients, we wished to assess whether immunosuppressants led to changes in the IM and whether these changes could be correlated with hyperglycemia. In particular, 16S characterization after immunosuppression showed interesting changes that were more investigated in depth using a metagenomics approach. This technology enabled the identification and functional characterization of bacterial species differentially enriched in immunosuppression. Additionally, metagenomic analysis permitted identification of differences in genes associated with diabetes in the context of immunosuppression. This study represents the first to characterize the IM in the context of immunosuppression at the metagenomic level, and provides interesting insight into a possible contribution to the diabetic phenotype in a large proportion of SOT recipients.

## Results

### Tacrolimus and Sirolimus-induced Hyperglycemia and Insulin Resistance

**Figure 1:**
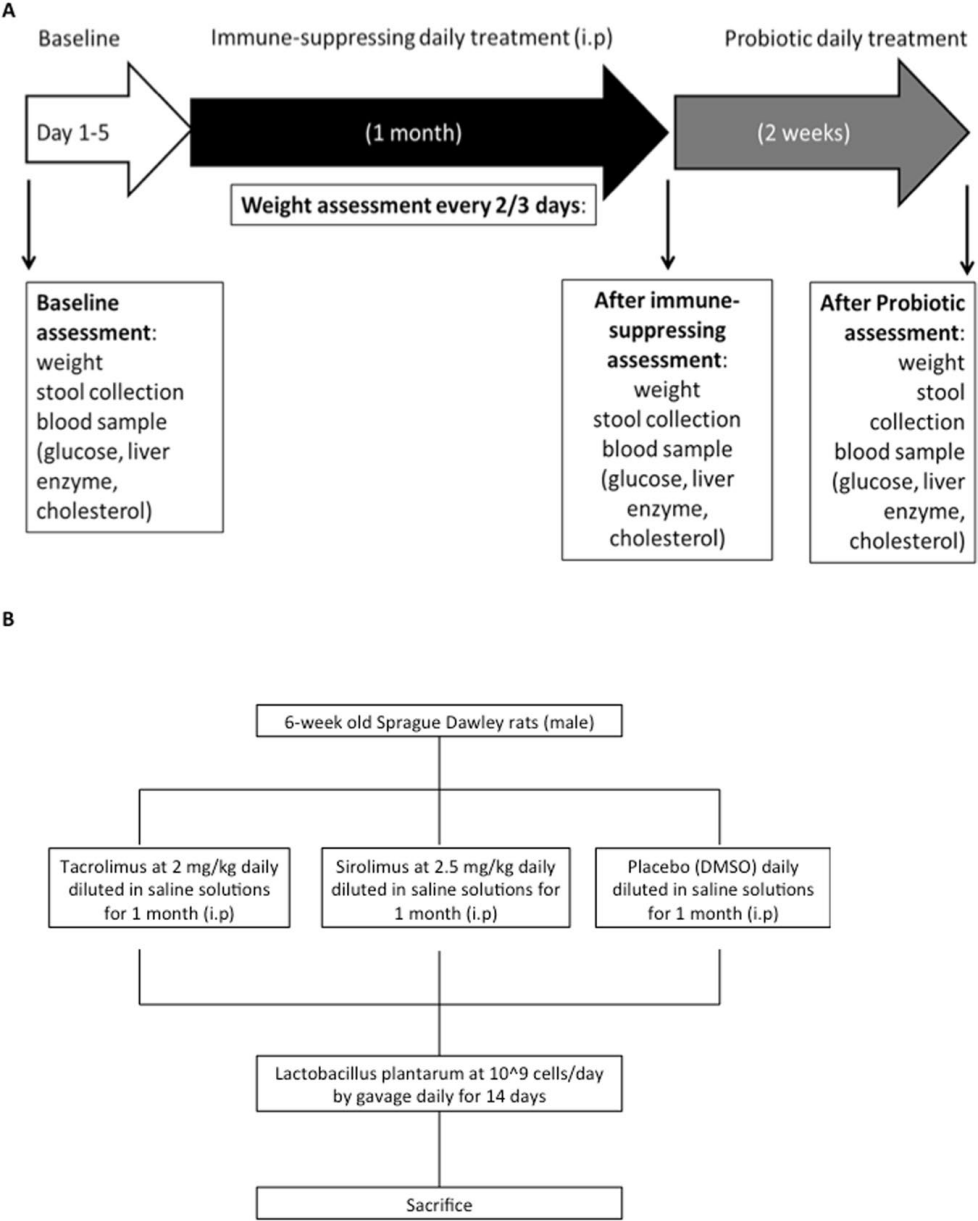
(**A**) Flow chart of the animal study evaluating the effects of immunosuppression on the intestinal microbiome; (**B**) Details regarding immunosuppression exposure in the timeline.

**Figure 2:**
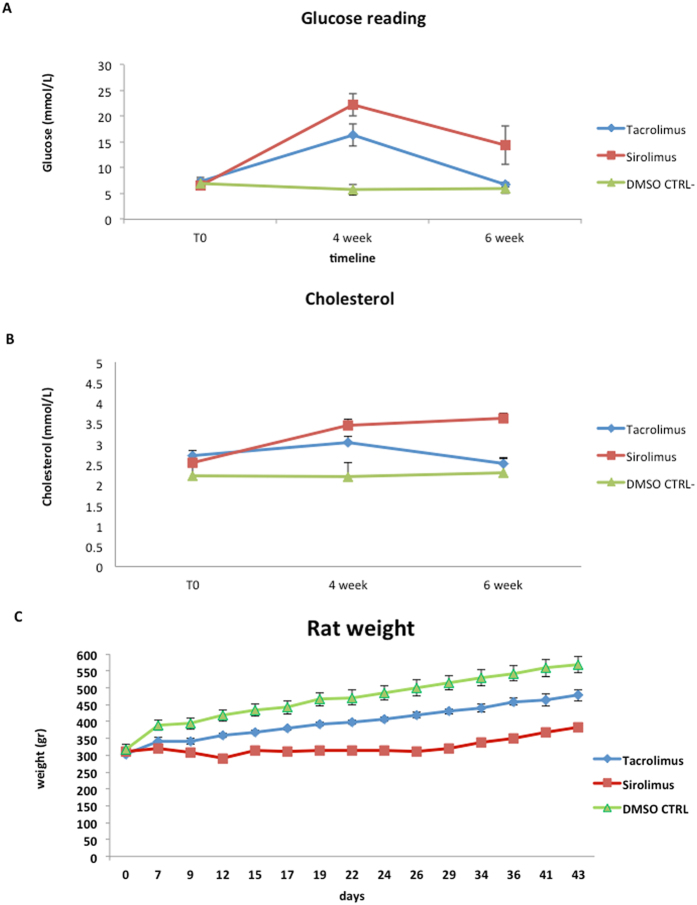
(**A**) Glucose levels were measured at all three time points, and mean values (±2.1 SEM) are reported at indicated time points. The values refer to 5 rats treated with Tacrolimus or Sirolimus and 4 with DMSO (P < 0.05 calculated with Student’s T-test analysis was considered statistically significant). (**B**) Cholesterol levels were measured at all three time points and mean values (±0.2 SEM) are reported at indicated time points. The values refer to 5 rats treated with Tacrolimus or Sirolimus and 4 animals with DMSO (P < 0.05 calculated with Student’s T-test analysis was considered statistically significant). (**C**) Rat weight assessment was done at baseline (day 0) every 3 days during the whole study, day 29 corresponds to after 1 month of immune suppression and day 43 is the final time point after probiotic treatment and mean values (±0.2 SEM) are reported at indicated time points. The values refer to 5 rats treated with Tacrolimus or Sirolimus and 4 animals with DMSO (P < 0.05 calculated with Student’s T-test analysis was considered statistically significant).

The flowchart of our animal study protocol is illustrated in Fig. 1A and B, and described in detail in the Methods section. Rats in both immunosuppressant groups developed significant hyperglycemia, with a glucose of 22 mmol/L (±2.1 SEM) in the Sirolimus group and 16 mmol/L (±2.1 SEM) in the Tacrolimus group (Fig. 2A), as compared to the normal glucose levels of 5.7 mmol/L in the control group (p = 0.0006). Glucose levels returned to normal in the Tacrolimus group following treatment with probiotics (6 mmol/L ± 0.4), whereas they decreased but remained elevated in the Sirolimus group (15 mmol/L, ±3.7). Cholesterol levels increased significantly by 4 weeks in both immunosuppressant groups (mean 3.5 mmol/L ± 0.3 in the Sirolimus group and mean 3.0 mmol/L ± 0.02 in the Tacrolimus group; p < 0.05 vs control) (Fig. 2B). The cholesterol normalized in the Tacrolimus group following probiotic therapy, but remained elevated in the Sirolimus group despite probiotics. The rate of weight gain was much slower in the Sirolimus group as compared to that in the Tacrolimus and control groups (Fig. 2C).

### 16S rRNA gene sequencing

**Figure 3:**
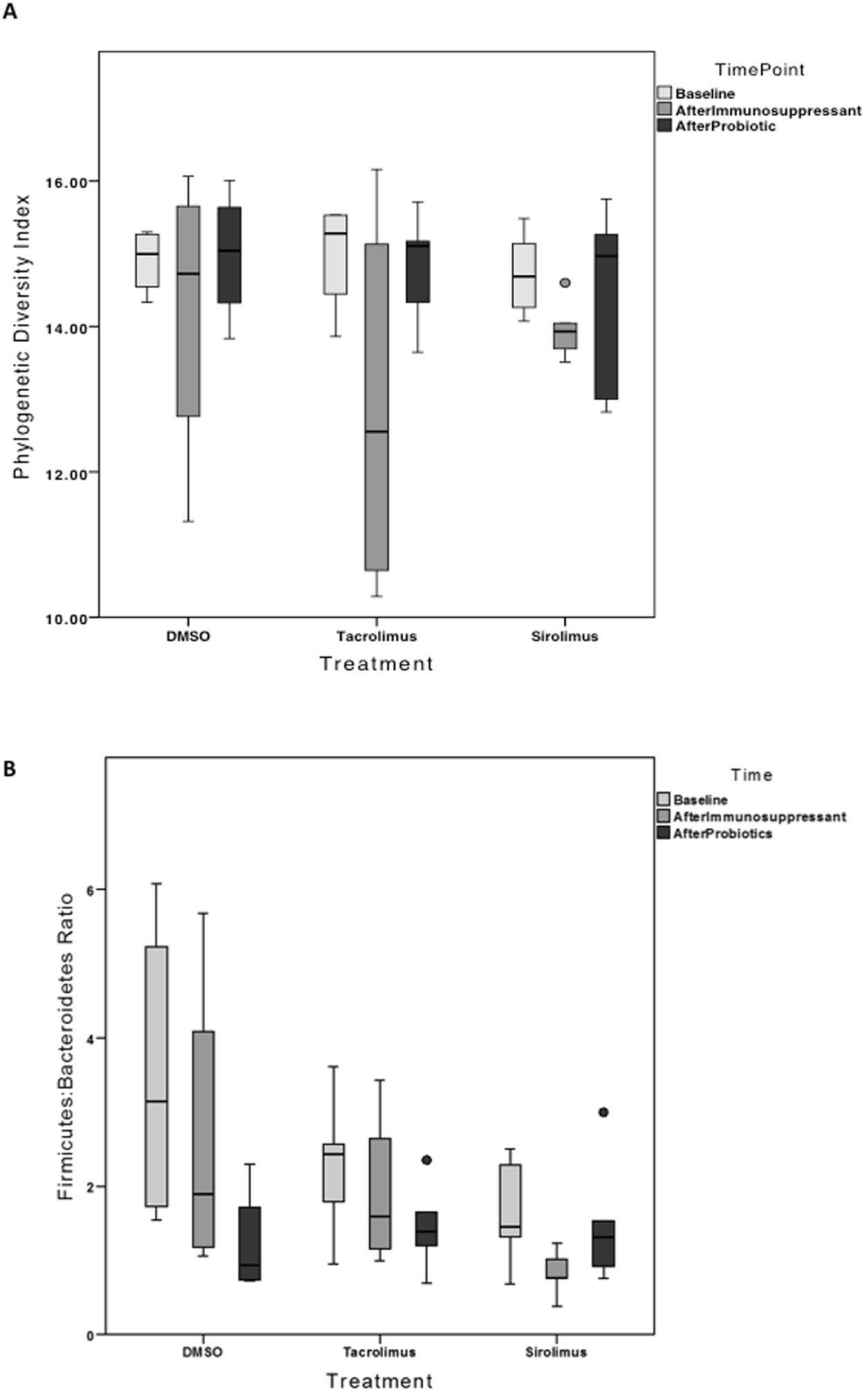
(**A**) Alpha diversities were compared by paired t-tests between all three treatment types (DMSO, Tacrolimus, Sirolimus), stratified by time points and no significant differences were observed. The values refer to 5 rats treated with Tacrolimus or Sirolimus and 4 animals with DMSO. Phylogenetic Diversity of rats in the Sirolimus treatment group was significantly lower after Sirolimus treatment (p < 0.05) than at baseline. (**B**) The relative abundance of Firmicutes and Bacteroidetes was determined for each sample and the ratio of Firmicutes/Bacteroidetes was calculated for samples at Baseline, After Immunosuppressant and After Probiotics for the treatments Tacrolimus, Sirolimus and the DMSO negative control. The values refer to 5 rats treated with Tacrolimus or Sirolimus and 4 animals with DMSO. There are no significant differences between the ratio of Firmicutes/Bacteroidetes across sampling time points or between different treatments. There is a decrease in this ratio for the Sirolimus treatment after immunosuppression, but it was not identified as significantly different (paired t-tests, p > 0.05).

**Figure 4:**
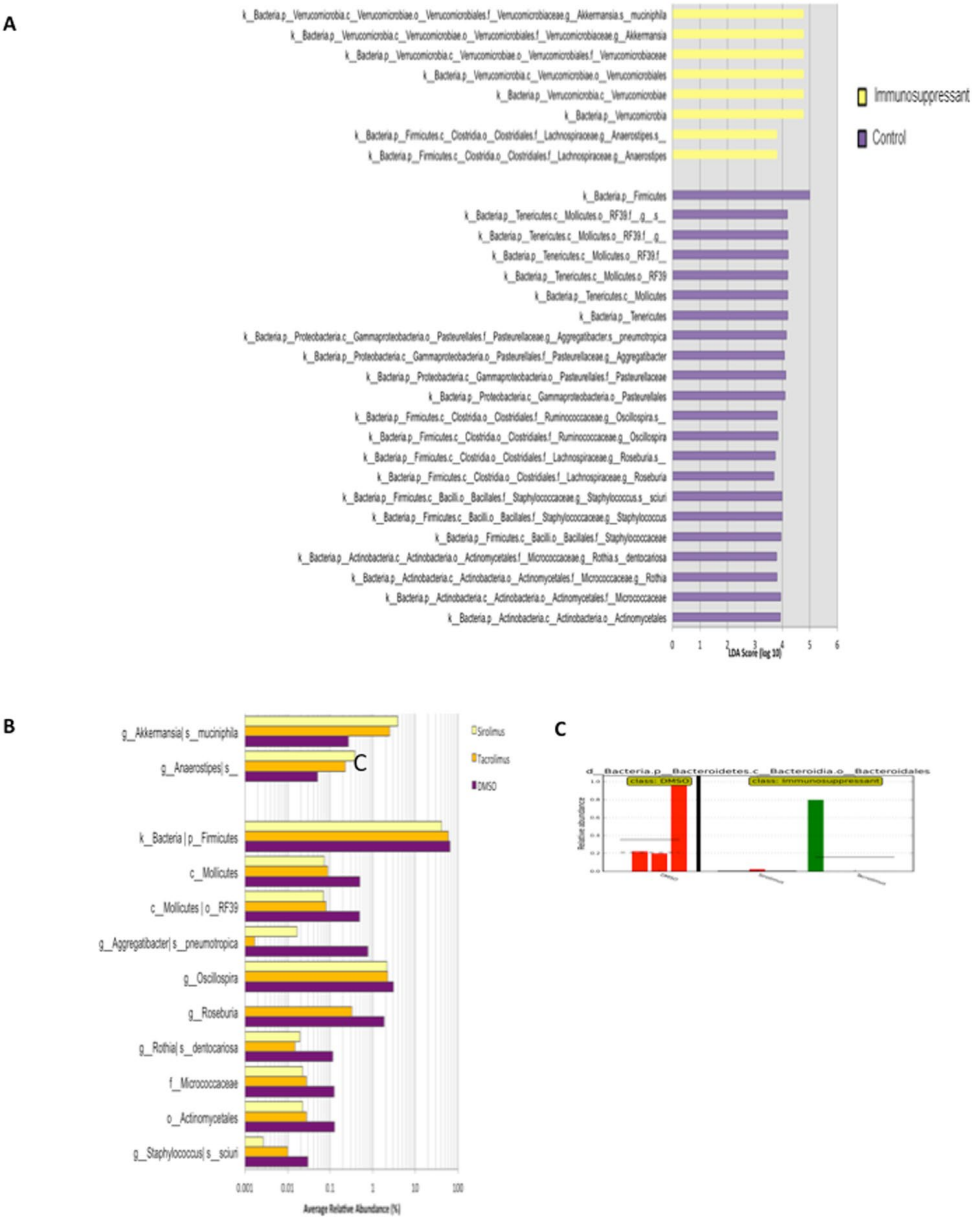
(**A**) The LDA effect size (LEfSe) analysis identified 30 OTU-level taxa with differential relative abundances between the DMSO (Control) group and the immunosuppressant treatment groups at time point 1 – after immunosuppressant treatment. A standard threshold of 2.0 logarithmic LDA score and an alpha value of 0.05 for the Kruskal-Wallis and paired Wilcoxon tests was used. The LDA scores of each of these significantly associated taxa are plotted, separated by the category that they are enriched in. The values refer to 5 rats treated with Tacrolimus or Sirolimus and 4 animals with DMSO. (**B**) The average relative abundances of these taxa with significant LDA scores are shown. Fewer taxa relative abundances are displayed than the total number of significant LDA scores, because of the redundant LDA scores when only one taxa is maintained at different taxonomic levels, resulting in significant associations at each level but overall being due to the same taxa. Taxa that were observed as significantly less abundant in immunosuppressant treatments include: *Roseburia, Oscillospira, Mollicutes, Rothia, Micrococcaceae, Actinomycetales* and *Staphylococcus*. (**C**) The IM composition of the immunosuppressed rats was no different from the control rats after treatment with probiotics. The only discriminating feature for the after probiotic time point was the taxa Bacteroidales being significantly enriched in DMSO treated rats compared to either of the immunosuppressive treatments. The values refer to 5 rats treated with Tacrolimus or Sirolimus and 3 animals with DMSO.

Bacterial Operational Taxonomic Units (OTUs) were characterized from the rat stool through V4 16S rRNA gene sequencing. The bacterial diversity of each stool sample was quantified using a Phylogenetic Diversity (PD) score, describing the evenness, richness and overall phylogenetic distribution of total bacteria in the sample. Alpha diversities were compared between all three treatment types (DMSO, Tacrolimus, Sirolimus), stratified by time point (Baseline, After Immunosuppressant, After Probiotic) and no significant differences were observed. Paired t-test were used to compare alpha diversities across the same rat within a treatment, between time points. A significant decrease in PD value was observed from baseline to after Sirolimus exposure (p < 0.05, Fig. 3A). Of note, the Firmicutes to Bacteroidetes ratio was not altered in either the Tacrolimus or Sirolimus groups. There was a decrease in this ratio after Sirolimus exposure, but it was not identified as significantly different (p > 0.05, Fig. 3B). The LDA effect size (LEfSe) analysis identified 30 OTU-level taxa with differential relative abundances between the DMSO (Control) group and the immunosuppressant treatment groups at time point 1 – after immunosuppressant treatment (Fig. 4A). Fewer taxa relative abundances are displayed than the total number of significant LDA scores, because of the redundant LDA scores when only one taxa is maintained at different taxonomic levels. This resulted in significant associations at each level, but overall being due to the same taxa. Taxa that were observed as significantly less abundant in immunosuppressant treatments include: Roseburia, Oscillospira, Mollicutes, Rothia, Micrococcaceae, Actinomycetales and Staphylococcus (Fig. 4B). Following treatment with probiotics, the IM composition of the immunosuppressed rats no longer had decreased abundance of the above taxa, and became similar to that of the control rats. The only discriminating feature for the after probiotic time point was the taxa Bacteroidales being significantly enriched in DMSO-treated rats compared to either of the immunosuppressive treatments.

### Metagenomic sequencing

**Figure 5:**
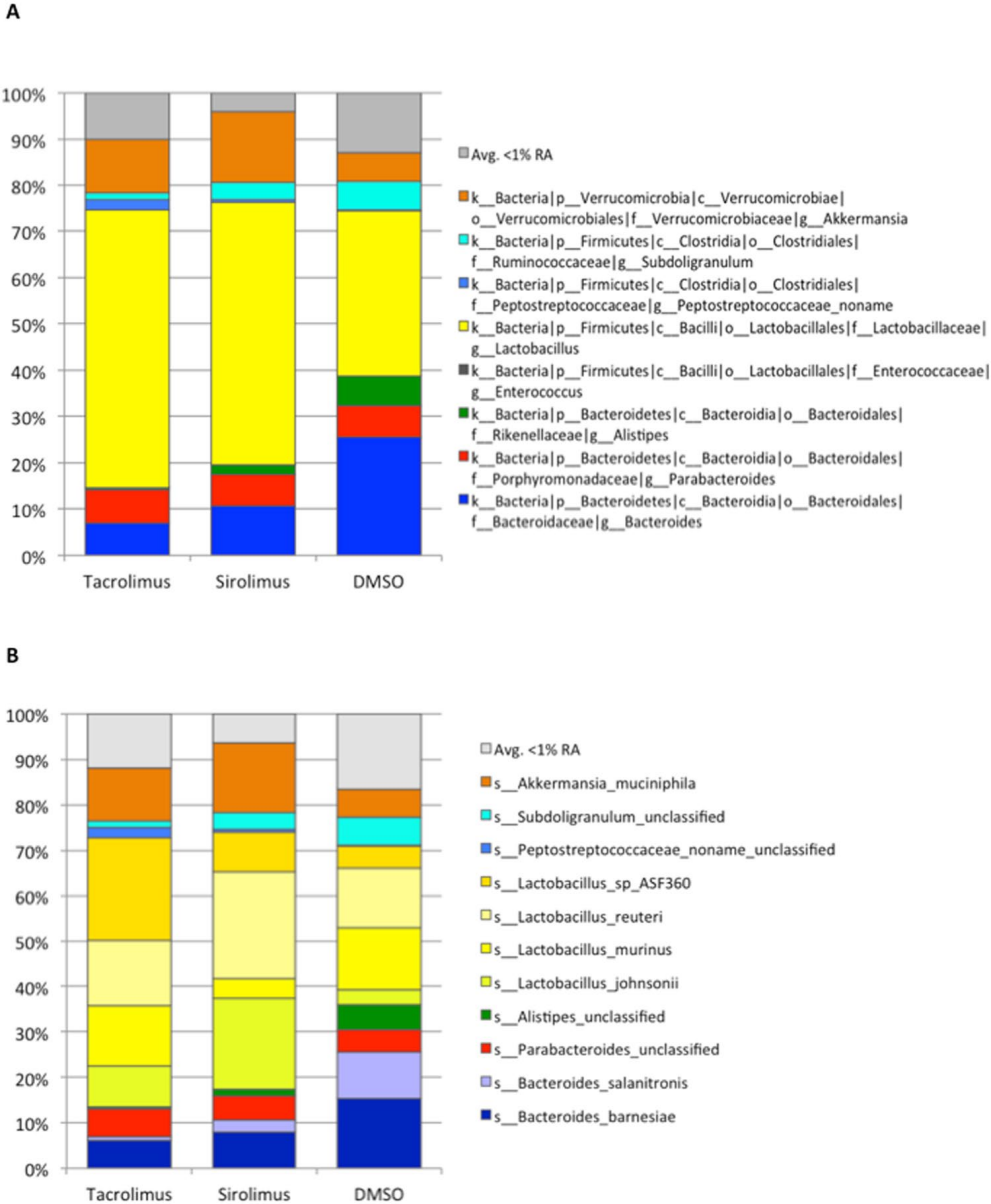
The relative abundances of (**A**) genera and (**B**) species identified in the metagenomic sequencing using MetaPhlAn taxonomic assignment method, grouped by treatment. Lactobacillus is the dominant genus in the community, and is composed of four species: *Lactobacillus sp. ASF360, reuteri, murinus* and *johnsonii*. Additionally, *Akkermansia muciniphila* was significantly more present in the microbiome of rats on immunosuppression (both tacrolimus and sirolimus). The values refer to 5 rats treated with Tacrolimus or Sirolimus and 3 animals with DMSO.

The relative abundances of genera identified using 16S V4 sequencing (data not shown) and metagenomic sequencing (Fig. 5A) showed similar overall trends in the samples. The relative abundance of species was determined through metagenomic sequencing of only the IM samples following immunosuppression exposure (Fig. 5B). This analysis was performed using the MetaPhlAn taxonomic assignment method, and identified Lactobacillus as the dominant genus in the community comprised of four species: *Lactobacillus sp. ASF360, reuteri, murinus and johnsonii* (Fig. 5B). *Akkermansia muciniphila* was another key species significantly more present in the IM of rats on immunosuppression (Fig. 5B).

### GO and KEGG ortholog database analysis

A pairwise Welch’s two-sided t-test was used to compare the control group to each treatment group, both separately by drug type and together. All the GO gene families specifically enriched in DMSO versus treatment group are represented for each comparison (Table S1). Only one GO family was significantly enriched in DMSO when the control group was compared to both immunosuppressant drugs. In particular, among the seven GO gene families enriched in treatment groups, 2 are commonly present in Sirolimus and Tacrolimus rats (Table S1).

**Figure 6:**
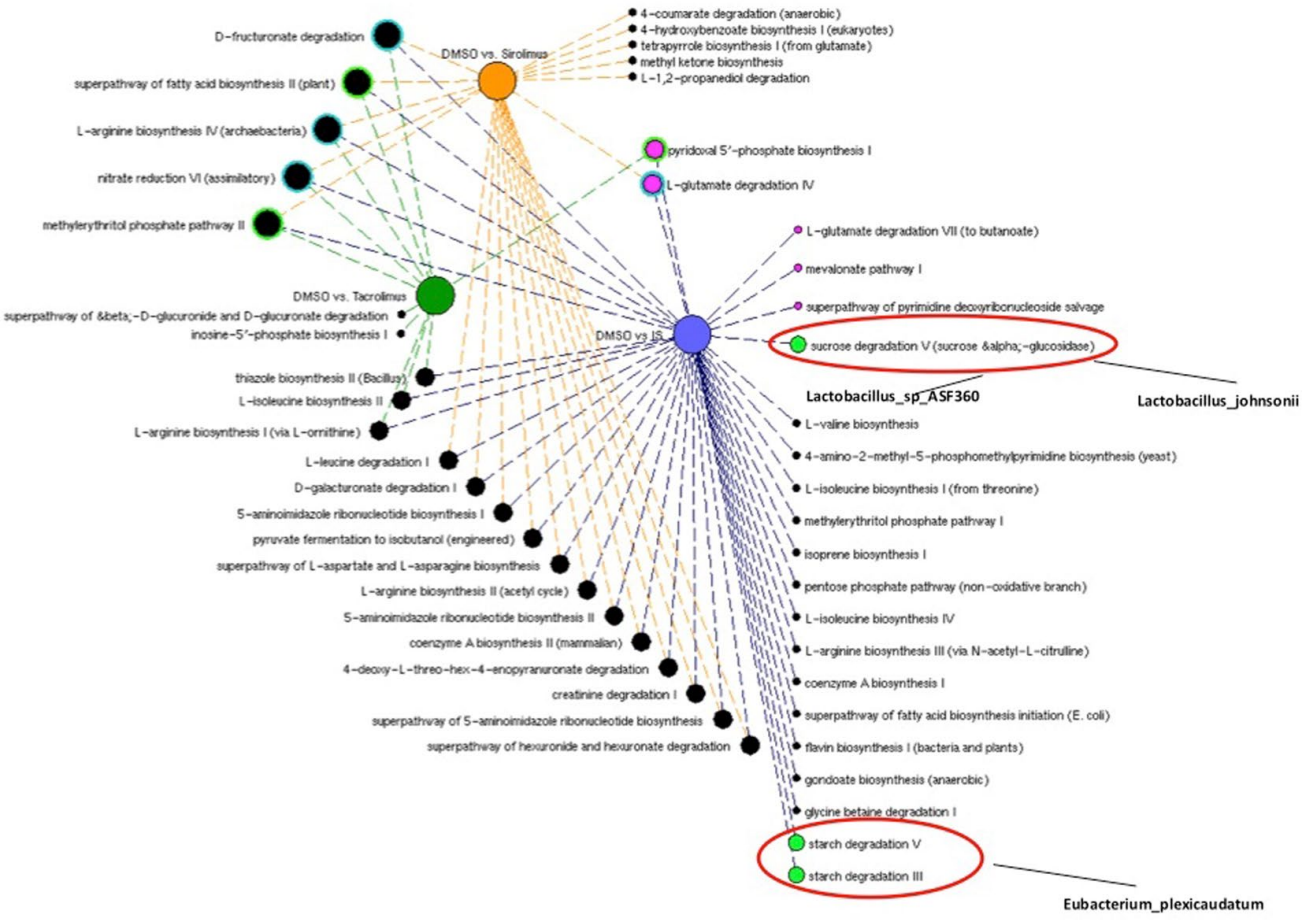
Network visualization of the metabolic pathways distinguishing the immunosuppressed rats (tacrolimus, sirolimus, and both treatment groups together) from the control rats using the Welch’s two-sided t-test (p < 0.05). DMSO and Sirolimus (orange), DMSO and Tacrolimus (green), and DMSO and both immunosuppressants –IS (purple) comparison were represented by nodes and the relative pathways abundances in black if enriched in control group or pink if enriched in treated groups. The size of the nodes is based on the degree (level of connectivity) of each node to the other nodes of the network. The most common pathways enriched in control or treated group are surrounded by a colored circle (blue if catabolic process or green if anabolic process). Three pathways involved in sucrose and starch metabolism were differentially enriched in the immunosuppressed versus control group. In particular, sucrose degradation V was enriched in immunosuppressant-treated rats. This is primarily due to the increased abundance of *Lactobacillus johnsonii* and Lactobacillus ASF360. Conversely, Starch degradation-related pathways are enriched in control rats. For the most part, the genetic origin cannot be identified and the taxonomy remains unclassified. The only taxa significantly contributing to the starch degradation V pathway was *Eubacterium plexicaudatum*.

**Figure 7:**
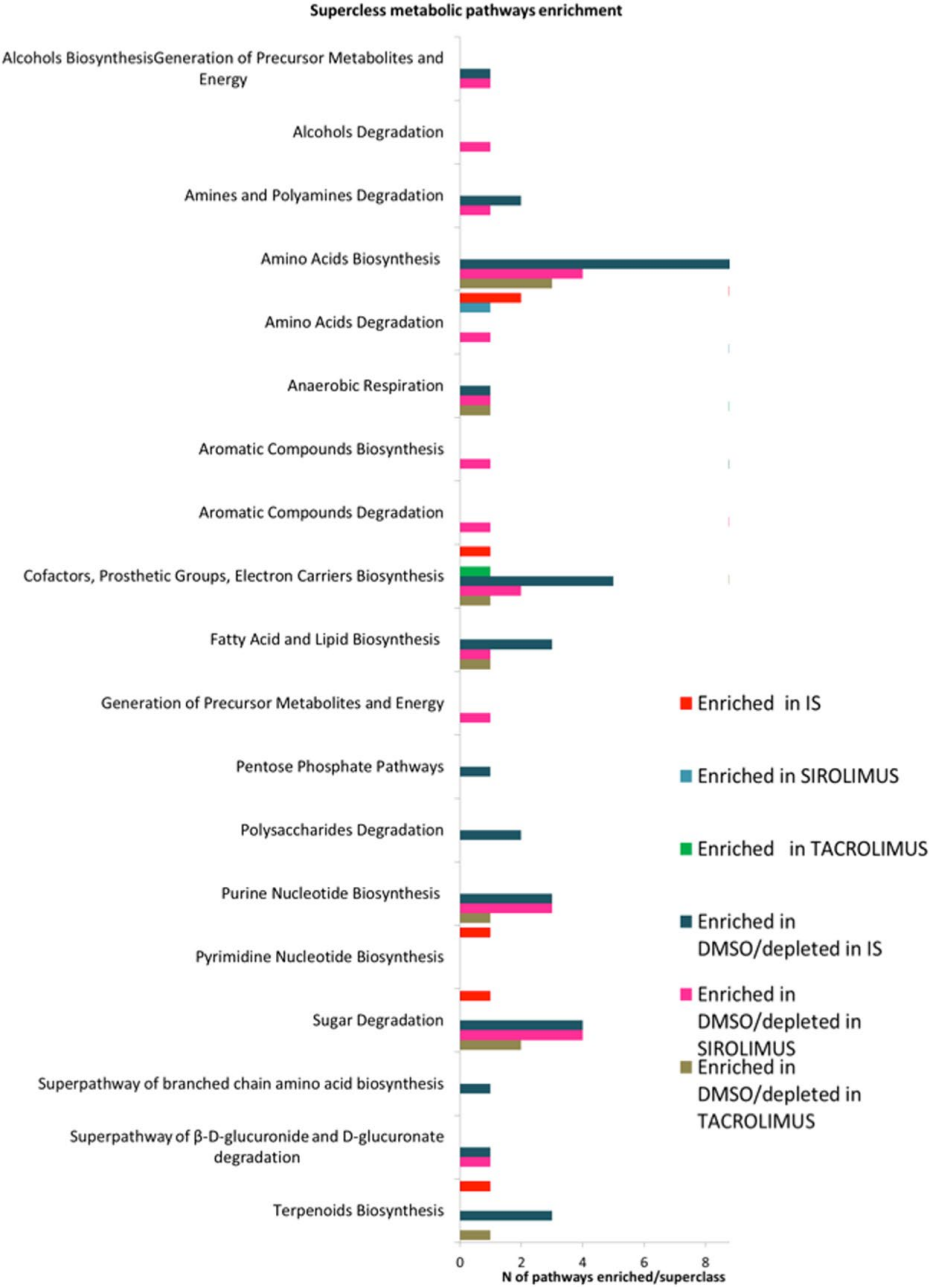
The chart shows the superclass distribution of the metabolic pathways enriched in the control (n = 3) or treated groups (n = 5).

**Figure 8:**
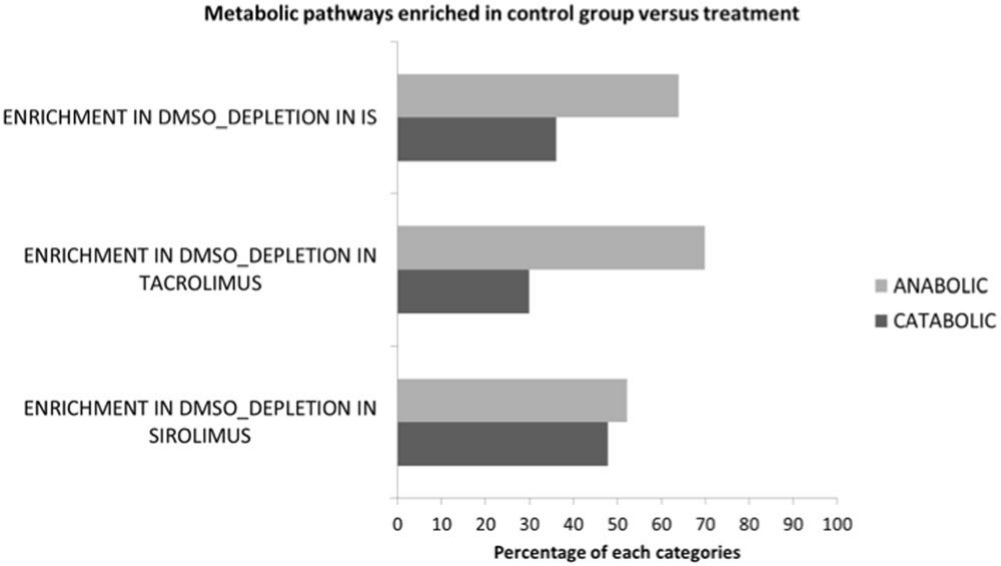
The percentage of catabolic and anabolic pathways enriched in control group (n = 3) compared to Sirolimus, Tacrolimus and both Immunosuppressant drugs (IS) (n = 5).

The metabolic pathways enriched in the different treatment groups, and indication as to anabolic and catabolic role, are listed in Table S2. The Tacrolimus and Sirolimus treatment groups had a greater number of catabolic than anabolic pathways compared to the control DMSO group. In order to visualize common pathway abundances enriched in immunosuppressed versus DMSO groups, we ran a network analysis (Fig. 6). The different comparisons were represented as colored nodes: DMSO and Sirolimus (orange), DMSO and Tacrolimus (green), DMSO and both immunosuppressants (purple), relative pathway abundances in black if enriched in control group, and pink if enriched in treated groups. The graphic visualization showed 5 pathways enriched in the control group and commonly present in all three of the comparisons. Among these five, three referred to anabolic processes (green circle surrounding the node) and 2 to catabolic pathways (blue circle). Two pathways were identified as commonly enriched in treated groups as presented in Fig. 6 (pink nodes) and Table S2. We then compared the immunosuppressant group as a whole (Tacrolimus and Sirolimus together) to DMSO control, which revealed a longer list of pathways (Table S3). Among those differentially enriched, Starch degradation-related pathways (Table S3) were significantly less abundant in immunosuppressant-treated rats as compared to control rats. The only taxa significantly contributing to starch degradation was *Eubacterium plexicaudatum* (Fig. 6). Conversely, genes coding for sucrose degradation were significantly enriched in the IM of immunosuppressant-treated rats (Table S3, Fig. 6). This is primarily due to the increased abundance of *Lactobacillus johnsonii* and *Lactobacillus ASF360* (synonymous with *Lactobacillus acidophilus*), which contribute almost entirely to all characterized sucrose degradation V genes in the metagenome. The metabolic Superclass pathways enriched or depleted in the various groups are represented in Fig. 7. The depletion of biosynthesis process is evident in immunosuppressed animals with a particular decrease in pathways involved in Amino Acid, Cofactor, Prosthetic Group, and Electron Carrier Biosynthesis. To elucidate the overall enrichment in catabolic or anabolic pathways for each group of rats, the relative percentage of anabolic and catabolic Superclasses in each comparison is reported in Fig. 8. This revealed an overall abundance in catabolic processes as opposed to anabolic processes in the immunosuppressant groups (Fig. 8), which is analogous to the IM composition in diabetes and obesity.

## Discussion

Metabolic complications after SOT are frequent, leading to a higher risk of cardiovascular complications and curtailed life expectancy^14, 15^. In this study, we demonstrate the distinct composition of the IM upon exposure to Tacrolimus and Sirolimus, highlighting how it is similar to that in diabetes and obesity. This pro-metabolic IM composition potentially contributes to the diabetic phenotype seen in a significant proportion of SOT recipients.

In our study, Tacrolimus- and Sirolimus-treated animals showed an alteration in taxa abundance similar to that previously reported in the IM of diabetes. In particular, Roseburia and Oscillospira were identified as less present in our treated rats, and are similarly known to be decreased in the IM of diabetic patients^6, 7^. These bacteria are butyrate-producing microorganisms that contribute to intestinal mucosal health. Interestingly, exogenous butyrate has been shown to improve insulin sensitivity systemically. Among the bacteria more represented in the IM of immunosuppressed rats, Lactobacillus was the dominant genus as highlighted by our metagenomic analysis. Another difference identified by metagenomic analysis was the abundance of *Akkermansia muciniphila* significantly more present in the IM of rats on immunosuppression, which is also characteristic of obese and diabetic patients.

In order to understand the implications of this change in IM composition, a pathway analysis was performed on the metagenomic data. This revealed an overall depletion of metabolic pathways in immunosuppression, similar to the pattern previously observed in patients with insulin resistance^16^. Additionally, our study reveals that genes involved in sucrose degradation are more commonly prevalent in the IM of immunosuppressed animals. Conversely, starch degradation-related pathways are significantly less represented in immunosuppressant-treated animals. These changes in bacterial gene composition are similar to those seen in diabetes^6^. *Eubacterium plexicaudatum*, known for its ability to digest saccharides and produce butyrate, was the only taxon to contribute significantly to starch degradation in control rats^17^. This taxon was less present in the immunosuppressed rats, thus depriving them of this butyrate-producing bacterium’s positive effect on the gut^16^. *Lactobacillus johnsonii* and *Lactobacillus ASF360* contributed almost entirely to all characterized sucrose degradation genes enriched in immunosuppressed rats by metagenomic sequencing. Sucrose degradation by the IM has already been shown to have a diabetogenic role in humans^18^, potentially contributing to the increment in glucose concentrations observed in our immunosuppressant-treated rats. Two putative mechanisms accounting for hyperglycemia in our immunosuppressed rats include a higher number of sucrose degradation genes in the IM, and an enrichment in bacteria able to quickly metabolize sucrose such as *Lactobacillus* ^19^. Manipulation of enteric flora with probiotics such as *Lactobacillus plantarum* has been shown to enhance growth of beneficial organisms, increases satiety, and decreases postprandial glucose excursions^20–23^. In our study, we exposed the animals to the probiotic Lactobacillus plantarum after the immunosuppression phase, and were able to demonstrate normalization of glucose in the tacrolimus group and improvement in the sirolimus group. However, the cholesterol levels normalized only in the tacrolimus group but not in the sirolimus group. In fact, Lactobacillus plantarum 299 V has demonstrated conflicting results with respect to decrease in cholesterol levels. In a study involving 36 healthy volunteers, Bukowska *et al*. found that the consumption of *Lactobacillus plantarum* 299 v did not contribute to changes in total serum cholesterol after 6 weeks^1^, although LDL cholesterol levels did decrease by 12%^22^. A meta-analysis on the effect of probiotics on lipid profiles has revealed that probiotics can improve cholesterol levels in mild hypercholesterolemia when there is a sustained period of exposure of >4 weeks, which we did not perform as it was hyperglycemia that was rather the primary endpoint of interest^23^. In our study, the tacrolimus group did not develop cholesterol levels as elevated as those in the sirolimus group. This may account for the difference in response to probiotics, with the tacrolimus group having normalization of cholesterol levels as opposed to the persistent hypercholesterolemia in the sirolimus group. Probiotics could therefore improve diabetes and obesity in organ transplant patients by altering the IM in a positive manner.

Few patient studies have been performed to characterize the IM in the setting of solid organ transplantation^24, 25^. These were short-term studies designed to assess for correlations with endpoints such as acute rejection^24^, with characterization only at the taxa level through 16S rRNA sequencing. Patients with acute rejection had a higher abundance of specific bacteria including Lactobacillales, *Enterococcus, Anaerofilum* and *Clostridium tertium* ^24^. These patients also had a lower abundance of *Ruminococcus* and *Bacteroides*. However, one must note that these patients were on antibiotic prophylaxis against *Pneumocystis jiroveci*, which affects the IM composition and may confound the interpretation of results. Specifically, those with acute rejection had been treated with multiple antibiotics for infections prior, which would further influence IM characterization. Additionally, these patients were at least on dual immunosuppressive therapy with prednisone and a calcineurin inhibitor, and occasionally on mycophenolate mofetil. A recent animal study assessing the effect of immunosuppressants on the IM used 16S rRNA sequencing, and did not find any differences at the taxa level^26^. Combined immunosuppressive treatment was shown to hinder innate antimicrobial defenses and enable overgrowth of commensal *E. coli* ^26^. The advantage of our animal study was the ability to study the effect of monotherapy on the IM composition, as well as detailed metagenomic sequencing to determine whether any molecular basis for metabolic complications after transplant could be discerned. We additionally found that probiotics can help in correcting immunosuppressant-associated enteric dysbiosis, reverting the IM composition back to control, which is also an important finding with clinical implications for transplant patients. We have thereby characterized the IM at the species level and the genes present in immunosuppression monotherapy conditions for the first time, which has key implications for the short- and long-term complications seen in all SOT recipients.

Our study admittedly has certain limitations. This was an animal study with a defined period of exposure to immunosuppression, whereas patients receive lifelong immunosuppression. Animal studies have been frequently used to evaluate changes in the IM under different conditions, and conclusions with important implications for patient care have been drawn from such studies. We could not obtain sufficient blood samples at the post-immunosuppression time point to check tacrolimus and insulin levels. Nonetheless, we employed an animal model with intraperitoneal injections of immunosuppressants known to develop hyperglycemia and insulin resistance with a dependable level of immunosuppression, similar to the human condition^27–29^. The probiotic treatment was attempted only as a proof-of-concept, hence immunosuppression was not maintained during this phase of the experiment. Therefore, it is possible that glucose levels returned to normal in the tacrolimus group because of stopping the tacrolimus. However, it should be noted that despite discontinuation of both immunosuppressants and initiation of probiotics, glucose levels only normalized in the tacrolimus but not sirolimus group, suggestive of a beneficial effect of the probiotic specifically in the former group. In future studies, butyrate supplementation could also be tried to re-establish the normal state of the intestinal microbiome.

Immunosuppressants often lead to the development of long-term complications after organ transplantation such as diabetes and obesity. The metabolic phenotype significantly impacts the long-term morbidity and mortality of SOT patients, and any measure to improve these outcomes is welcome. An appreciation of the IM distribution and bacterial gene composition under the effect of immunosuppressants is helpful in the development of a personalized approach to treatment of diabetes in this context. Future study to evaluate the IM solid organ transplant recipients is ongoing, and will pave the way to considering probiotics or prebiotics to restore a microbial balance that is more conducive to health.

## Methods

### Animals

Five-week-old male Sprague-Dawley® rats were purchased from Charles River, and were kept at the animal facility at the Toronto General Hospital Research Institute. They were housed in sterilized, individually-ventilated cages on autoclaved corn cob bedding with free access to acidified water through an automatic watering system and autoclaved rodent diet (Teklad LM-485, Harlan Teklad, Indianapolis, IN) on a 12:12-h light-dark cycle. The humidity was maintained between 40–70% and temperature between 20–22 °C. The animal care and use program is certified by the Canadian Council on Animal Care, and all procedures were approved by the local institutional Toronto General Research Institute Animal Care Committee. All experiments were performed in accordance with the relevant guidelines and regulations. The rats were acclimated for 1 week, following which they were assigned to three experimental arms: intraperitoneal injections of (1) Tacrolimus at 2 mg/kg daily (n = 5 rats), (2) Sirolimus at 2.5 mg/kg daily (n = 5 rats), (3) control with DMSO injections (n = 4 rats) for 28 days^18, 29^. These medications were reconstituted in saline for a total volume of 0.2 mL. Following the 4-week immunosuppression phase, probiotic (Lactobacillus plantarum at 1 × 10^9^ cells) was given via gavage daily for a 2-week period. Rat food intake was monitored on a daily basis, and was the same across the different conditions. Weights were recorded every 2 days. The flowchart of this protocol is illustrated in Fig. 1A and B. The animal study and the relative experiments were performed once.

### Sample collection and processing

Blood and stool samples were obtained at baseline, after immunosuppression and after probiotic exposure. 1 mL of whole blood was collected from the saphenous vein, and serum was spun down, in order to obtain transaminase, cholesterol, glucose and albumin levels using VetScan VS2 (Abaxis, USA). Fresh fecal pellets from the rats were obtained at the appropriate time points, and immediately snap-frozen and stored at −80C. At the end of the 6 weeks, rats were euthanized by exsanguination under isoflurane anesthesia. Significant differences for weights, glucose and cholesterol levels between the two groups were calculated using Student’s T-test analysis (P < 0.05 was considered statistically significant) using online software Graph Prism (http://www.graphpad.com/scientific-software/prism/).

### Stool DNA extraction

DNA was extracted from 200 mg of stool pellets using E.Z.N.A.® Stool DNA Kit (Omega BioTek) following manufacture’s protocol. The final yield and quality of samples were assessed by NanoDrop ND-1000 UV-Vis Spectrophotometer.

### 16S rRNA V4 Region Gene Sequencing and Analysis

The V4 hypervariable region of the 16S rRNA gene was amplified using a universal forward sequencing primer and a uniquely barcoded reverse sequencing primer to allow for multiplexing^30^. All amplification reactions were done in triplicate, and pooled to reduce amplification bias. The final library was purified with 0.8X Agencourt Ampure XP magnetic beads, quantified and loaded on to the Illumina MiSeq for sequencing, according to manufacturer instructions (Illumina, San Diego, CA).

The UPARSE pipeline, available through USERACH, was used for sequence analysis^31, 32^. Sequences were clustered into operational taxonomic units (OTUs) at 97% identity. Chimeras were detected and removed using the –uchime_ref reference based method and the Ribosomal Database Project (RDP) 16S database, derived from the RDP training set version 9, accessed through USEARCH^33^. Assembled sequences were mapped back to the chimera-free OTUs. Taxonomy assignment was determined using QIIME with default methods and the GreenGenes v. 13.8 database^34^. OTU sequences were aligned using PyNast accessed through QIIME, and a phylogenetic tree of the aligned sequence data was made using FastTree^35^.

Low abundance OTUs (<0.005% RA) were removed from the analysis^36^. Relative abundances of the community members were determined using the rarefied data and summarized at each taxonomic level. Alpha and beta diversity were calculated using the rarefied OTU-level data in QIIME^34^. Weighted and unweighted UniFrac distances were calculated and visualized using Principal Coordinates Analysis (PCoA) in EMPeror^21, 34^. Statistical analyses were performed using SPSS Statistics version 20.0.0 (IBM, United States). LEfSe analysis were calculated on the Huttenhower Galaxy server^37^.

### Metagenomic Library Prep, Sequencing and Analysis

Samples from the After Immunosuppression time point were selected from the dataset for metagenomic sequencing. Fragmented DNA was prepared for sequencing using the NEBNext DNA Library Prep Master Mix set (New England BioLabs) and Illumina-based homebrew adapters, following the standard protocol of end-repair, dA-tailing and adapter ligation. The library was amplified following the NEBNext DNA Library Prep Master Mix set protocol and quantified using the Agilent BioAnalyzer and even quantities of each sample were pooled to create a final library for sequencing. The metagenomic library was sequenced on the Illumina NextSeq Data was converted from bcl format, output from the NextSeq, to FASTQ using the bcl2fastq2 Illumina conversion software.

The HUMAnN2 pipeline was used to analyze the metagenome of each sample^38^. Quality-filtered DNA reads were input into the pipeline, and a taxonomic profile was created using MetaPhlAn2 and the ChocoPhlAn pangenome database^39^. The pangenome was then mapped and associated with the taxonomic profile using Bowtie 2, creating a list of organism-specific gene hits^40^. The result of the HUMAnN2 pipeline is gene family abundances, pathway abundances and pathway coverage, all stratified by organism. These results were compared between the categories of interest to identify enriched and depleted pathway abundances as well as gene families using the STAMP software package^41^ available online http://kiwi.cs.dal.ca/Software/STAMP. We did identify one sample in the control group as being an outlier based on Principal Component Analysis, and thus excluded it from the analysis. A Welch’s two-sided t-test was used to compare two treatment groups (DMSO and Sirolimus, DMSO and Tacrolimus, and DMSO and both immunosuppressants) with a p-value set at <0.05.

### Network Construction

All the enriched pathway abundances and relative categories of treatment with a significant p value < 0.05 were uploaded into NAViGaTOR 2.3, a graphing application for biological networks available online (http://ophid.utoronto.ca/navigator)^42^. All the enriched pathway abundances in the different treatment comparisons were represented by nodes linked with edges to the three different treatment comparison (DMSO and Sirolimus, DMSO and Tacrolimus, DMSO and both immunosuppressants).

## Electronic supplementary material

Supplementary information
