# Supplementary material for: Impact of Immunosuppression on the Metagenomic Composition of the Intestinal Microbiome: a Systems Biology Approach to Post-Transplant Diabetes
Source: Sci Rep. 2017 Aug 31;7:10277. doi: 10.1038/s41598-017-10471-2 (PMC5578994; doi:10.1038/s41598-017-10471-2)

**Supplementary Table 1:** GO gene families identified by Welch’s two-sided t-test (p<0.05) as enriched in DMSO and Sirolimus, DMSO and Tacrolimus, and DMSO and both immunosuppressants comparisons are listed.

**Supplementary Table 2:** Pathways identified as enriched in the two different comparisons (DMSO and Sirolimus, DMSO and Tacrolimus) by the Welch’s two-sided t-test (p<0.05) are listed. They are divided in the 2 different comparisons considered in the analysis and grouped by the type of metabolic process (catabolic or anabolic). All the pathways enriched in the treated group are in bold font.

**Supplementary Table 3:** Pathways identified as enriched in the DMSO and immune suppressive drugs comparison by the Welch’s two-sided t-test (p<0.05) are listed. They are grouped by the type of metabolic process (catabolic or anabolic). All the pathways enriched in the treated group are in bold font.

**Supplementary Figure 1** Welch’s two-sided t-test was performed to compare the relative abundances of different species between DMSO and sirolimus treatment, DMSO and tacrolimus treatment and between tacrolimus and sirolimus treatments. Only between DMSO and sirolimus treatment were any species relative abundances identified as significantly different. These species were Lactobacillus johnsonii and *Mucispirillum schaedleri*, that were both found to be significantly enriched in sirolimus treated rats ( p < 0.01).


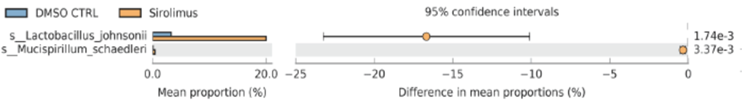

Supplement: Supplementary file 1 — Supplementary information [file 41598_2017_10471_MOESM1_ESM.docx]
